# Does the Superior Septal Approach Increase the Incidence of Postoperative Junctional Rhythm Compared to the Right-Sided Left Atriotomy?: A Comparison in Minimally Invasive Mitral Valve Surgery via Right Mini-Thoracotomy

**DOI:** 10.5761/atcs.oa.25-00095

**Published:** 2025-08-14

**Authors:** Masataka Yamazaki, Yorihiko Matsumoto, Tatsuo Takahashi, Hirofumi Haida, Naritaka Kimura, Kenichi Hashizume, Hideyuki Shimizu

**Affiliations:** Department of Cardiovascular Surgery, Keio University, School of Medicine, Tokyo, Japan

**Keywords:** minimally invasive mitral valve surgery, drawer-case technique, superior septal approach, junctional rhythm

## Abstract

**Purpose:** The superior septal approach offers improved mitral valve exposure compared to the right-sided left atriotomy or transseptal approach. However, the risk of postoperative sinus node dysfunction remains controversial, with limited data in the context of right mini-thoracotomy.

**Methods:** This retrospective study included 155 patients (64 women; mean age, 60.8 ± 13.4 years) who underwent mitral valve surgery via right mini-thoracotomy between November 2016 and August 2023. Indications included degenerative mitral regurgitation (94.8%) and mitral stenosis (5.2%). Patients were divided into 2 groups: the conventional minimally invasive mitral valve surgery (CM) group (n = 47), using the right-sided left atriotomy, and the drawer-case technique (DCT) group (n = 108), using the superior septal approach. Demographic, intraoperative, and outcome data were analyzed.

**Results:** Baseline characteristics were similar between groups. There were no significant differences in valve repair techniques or postoperative echocardiographic findings. Postoperative junctional rhythm occurred in 6 patients (CM group) and 21 patients (DCT group); all patients with preoperative sinus rhythm returned to sinus rhythm postoperatively.

**Conclusion:** The superior septal approach does not increase the risk of persistent junctional rhythm in right mini-thoracotomy and is a safe and effective option for mitral valve surgery.

## Introduction

Since minimally invasive mitral valve surgery (MIMVS) is generally performed via a right mini-thoracotomy, a right-sided left atriotomy is anatomically considered the optimal approach for direct access to the left atrium.^1,2)^ However, in cases where the left atrium is small or the heart is rotated counterclockwise, it may be challenging to obtain an adequate surgical view through a right-sided left atriotomy. Furthermore, the use of an atrial retractor placed on the roof of the left atrium may further displace the mitral valve toward the bottom of a “well,” making complex procedures more difficult.^3)^ In light of these challenges, we have reported the advantages of using a superior septal approach for mitral valve access through a right mini-thoracotomy.^4)^ However, the superior septal approach is associated with risks such as the development of junctional rhythm and the potential need for permanent pacemaker implantation.^5–7)^ Therefore, in patients undergoing mitral valve surgery via right mini-thoracotomy, we compared and analyzed perioperative factors—including the incidence of postoperative junctional rhythm and the number of cases requiring permanent pacemaker implantation—between the right-sided left atriotomy and the superior septal approach.

## Materials and Methods

### Drawer-case technique (DCT)

**Fig. 1:**
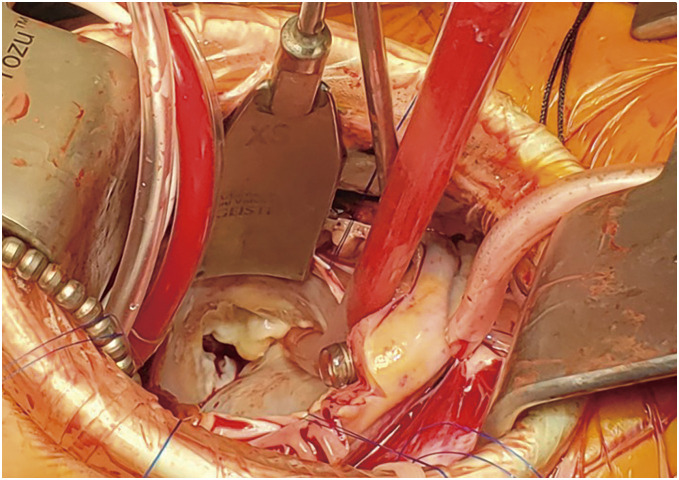
The mitral valve exposed by the DCT approach is in close proximity to the chest wall and is accessible within finger’s reach. DCT: drawer-case technique

A main feature of the DCT is the visual field development technique with several pericardial traction sutures. The pericardial retraction suture on the diaphragmatic side stretches the vertical axis from the superior vena cava to the inferior vena cava. The mitral valve is exposed via a superior septal approach, and other traction sutures allow the valve to be brought closer to the right chest wall wound and ensure a substantial working space around the valve by guiding the pericardial retraction sutures from outside the wound using an Endo Close needle-suture device (Covidien Inc., Minneapolis, MN, USA). This approach helps surgeons achieve a surgeon-friendly visual field that surpasses the median sternotomy provided in mitral valve surgery (**Fig. 1**). The details of this method have been previously reported.^4)^ We named this technique the DCT because it resembles looking into a desk drawer from above.

### Patients and study design

Our institutional ethics committee approved the study protocol (reference number: 20150123), and informed consent was obtained from each patient. We included 155 patients (64 women; mean age, 60.8 ± 13.4 years) who underwent mitral valve surgery through a right mini-thoracotomy as the initial surgical therapy from November 2016 to August 2023. The indications for surgery were degenerative mitral valve regurgitation (147 patients, 94.8%) and mitral valve stenosis (8 patients, 5.2%). Patients with reduced cardiac function (ejection fraction ≤40%), significant aortic plaque, a dilated ascending aortic diameter of ≥40 mm, severe thoracic deformity, significant adhesions in the right thoracic cavity, or extreme left shift of the heart were excluded from the study. The 155 patients were divided into 2 groups: those who underwent mitral valve surgery via the right-sided left atriotomy approach (conventional MIMVS group, n = 47) and those who underwent mitral valve surgery via the superior septal approach (DCT group, n = 108). The operative procedure was chosen according to the surgeon’s preference. Both groups’ demographic, intraoperative, and outcome data were collected retrospectively and analyzed through descriptive statistics.

### Statistical analysis

Descriptive statistics (arithmetic mean, median if indicated, minimum and maximum, and standard deviation) were calculated for continuous variables, whereas absolute frequencies and percentages were calculated for qualitative variables. A P-value of <0.05 was considered significant. Statistical analyses were performed using JMP 17 Statistical Software 2022 (SAS Institute Inc., Cary, NC, USA).

## Results

**Table 1** shows the patients’ baseline characteristics. Compared with the CM group, the DCT group contained a significantly higher proportion of advanced-age and female patients, and their body surface area was significantly smaller. The 2 groups had no significant differences regarding lesion site, valve repair techniques, or sternovertebral distance (S–V distance). The clinical outcomes of the patients who underwent mitral valve repair are described in **Table 2**. Among the 137 patients who underwent mitral valve repair, those in whom the repair was performed with leaflet resection alone accounted for the majority in both groups: 31 (66.0%) patients in the CM group and 53 (58.9%) patients in the DCT group. Autologous pericardium was used for posterior leaflet augmentation in 5 (10.6%) patients in the CM group and 9 (10.0%) patients in the DCT group, with no significant difference. Use of full rings was more common, and larger ring sizes were selected in the DCT group. There were no significant differences between the 2 groups in terms of postoperative transthoracic echocardiographic evaluation of regurgitation or the average pressure gradient difference across the mitral valve position.

**Table 1:** Baseline characteristics of the patients

Variables	Total (n = 155)	CM (n = 47)	DCT (n = 108)	*P* value
Age (year)	60.8 ± 13.4	56.2 ± 10.2	62.8 ± 14.2	0.005
Female (%)	64 (41.3)	11 (23.4)	53 (49.1)	0.003
BSA (m^2^)	1.63 ± 0.22	1.73 ± 0.20	1.58 ± 0.21	0.0001
Mitral stenosis (%)	8 (5.2)	1 (2.1)	7 (6.5)	0.26
Mitral regurgitation (%)	147 (94.8)	46 (97.9)	101 (93.5)	0.26
Degenerative mitral regurgitation (%)	148 (95.5)	46 (97.9)	101 (93.5)	0.26
Functional mitral regurgitation (%)	12 (7.7)	0 (0.0)	12 (11.1)	0.02
Lesion site				
Anterior leaflet (%)	15 (9.7)	5 (10.6)	10 (9.4)	0.8
Posterior leaflet (%)	80 (52.0)	28 (59.6)	52 (48.6)	0.21
Combination of anterior and posterior leaflets (%)	60 (38.7)	14 (29.8)	46 (42.6)	0.15
Atrial fibrillation (%)	35 (22.6)	9 (19.2)	26 (24.1)	0.5
Sternovertebral distance (mm)	102.9 ± 17.9	105.4 ± 18.3	101.8 ± 17.6	0.26
Sternovertebral distance <75 mm (%)	12 (7.7)	4 (8.5)	8 (7.4)	0.81
Sternovertebral distance <60 mm (%)	3 (1.9)	1 (2.1)	2 (1.9)	0.91

Values display total number of patients reported as mean ± SD.

BSA: body surface area; CM: conventional MIMVS; DCT: drawer-case technique

**Table 2:** Clinical outcomes of the patients who underwent mitral valve repair

Variables	Total (n = 137)	CM (n = 47)	DCT (n = 90)	*P* value
Leaflet resection (%)	84 (61.1)	31 (66.0)	53 (58.9)	0.42
Leaflet resection + artificial chordae (%)	44 (32.1)	20 (42.6)	24 (26.7)	0.04
Posterior mitral leaflet augmentation due to autologous pericardial patch (%)	14 (10.2)	5 (10.6)	9 (10.0)	0.87
Mitral annuloplasty				
Full ring (%)	28 (20.4)	3 (6.4)	25 (27.8)	0.004
Partial band (%)	108 (78.8)	43 (91.5)	65 (72.2)	0.004
Size of ring/band ≤30 mm (%)	44 (32.4)	20 (42.6)	24 (26.7)	0.04
2nd aortic cross-clamping due to residual mitral regurgitation (%)	6 (4.4)	3 (6.4)	3 (3.3)	0.31
Evaluation of mitral regurgitation by postoperative TTE				
None	43 (31.4)	12 (25.5)	31 (34.4)	0.29
Trivial	68 (49.6)	24 (51.1)	44 (48.9)	0.81
Mild	24 (17.5)	10 (21.3)	14 (15.6)	0.4
Mild–moderate	2 (1.5)	1 (2.1)	1 (1.1)	0.64
>Moderate	0 (0.0)	0 (0.0)	0 (0.0)	—
Mean mitral valve gradient (mmHg)	2.6 ± 1.1	2.5 ± 0.9	2.7 ± 1.2	0.35

Values display total number of patients reported as mean ± SD.

CM: conventional MIMVS; DCT: drawer-case technique; TTE: transthoracic echocardiography

The patients’ clinical outcomes are shown in **Table 3**. All 18 patients in whom mitral valve replacement was performed were in the DCT group. This reflects the fact that mitral valve replacement was not historically indicated for mitral valve surgery through a right mini-thoracotomy in our institute because of concern regarding the risk of left ventricular rupture. In recent years, mitral valve replacement has been included as an option for MIMVS through a right mini-thoracotomy. Concomitant procedures performed during mitral valve surgery included tricuspid annuloplasty in 69 (44.5%) patients, left atrial appendage closure in 39 (25.2%), pulmonary vein isolation in 17 (11.0%), the maze procedure in 11 (7.1%), left atrial plication in 6 (3.9%), aortic valve replacement (AVR) in 5 (3.2%), and Morrow myectomy and ventricular septal defect repair in one patient each. There were no differences between the 2 groups in terms of these concomitant procedures.

**Table 3:** Clinical outcomes

Variables	Total (n = 155)	CM (n = 47)	DCT (n = 108)	*P* value
Mitral valve replacement (%)	18 (11.6)	0 (0.0)	18 (16.7)	0.003
Mitral valve repair (%)	137 (88.4)	47 (100.0)	90 (83.3)	0.003
Cardiopulmonary bypass time (min)	213.2 ± 35.2	209.9 ± 36.6	214.7 ± 34.7	0.44
Aortic cross-clamping time (min)	145.7 ± 30.6	152.1 ± 32.2	143.0 ± 29.6	0.08
Antegrade cardioplegia for myocardial protection (%)	155 (100.0)	47 (100.0)	108 (100.0)	—
Retrograde cardioplegia for myocardial protection (%)	101 (65.2)	6 (12.8)	95 (88.0)	0.0001
Conversion to sternotomy (%)	0 (0.0)	0 (0.0)	0 (0.0)	—
Coronary sinus injury during retrograde myocardial protection (%)	3 (1.9)	0 (0.0)	3 (2.8)	0.25
Concomitant procedure				
Tricuspid valve annuloplasty (%)	69 (44.5)	17 (36.2)	52 (48.2)	0.17
Left atrial appendage closure (%)	39 (25.2)	8 (17.0)	31 (28.7)	0.12
Pulmonary vein isolation (%)	17 (11.0)	7 (14.9)	10 (9.3)	0.3
MAZE (%)	11 (7.1)	5 (10.6)	6 (5.6)	0.26
Left atrial plication (%)	6 (3.9)	0 (0.0)	6 (5.6)	0.09
Aortic valve replacement (%)	5 (3.2)	1 (2.1)	4 (3.7)	0.61
Ventricular septal defect closure (%)	1 (0.7)	0 (0.0)	1 (0.9)	0.51
Morrow myectomy (%)	1 (0.7)	0 (0.0)	1 (0.9)	0.51
Intensive-care unit stay (day)	2.0 ± 1.3	1.9 ± 1.4	2.0 ± 1.2	0.39
Congestive heart failure (%)	1 (0.7)	0 (0.0)	1 (0.9)	0.51
Eventration of diaphragm (%)	3 (1.9)	2 (4.3)	1 (0.9)	0.17
Intercostal neuralgia (%)	2 (1.3)	1 (2.1)	1 (0.9)	0.54
Cerebral infarction (%)	1 (0.7)	0 (0.0)	1 (0.9)	0.51
Re-expansion pulmonary edema (%)	1 (0.7)	1 (2.1)	0 (0.0)	0.13
Postoperative atrial fibrillation (%)	67 (43.2)	15 (31.9)	52 (48.2)	0.06
Postoperative junctional rhythm (%)	27 (17.4)	6 (12.8)	21 (19.4)	0.31
Permanent pacemaker implantation (%)	1 (0.7)	0 (0.0)	1 (0.9)	0.51
Atrial fibrillation at discharge (%)	19 (12.3)	3 (6.4)	16 (14.8)	0.43
Sinus rhythm at discharge (%)	135 (87.1)	44 (93.6)	91 (84.3)	0.43
Inhospital stay (day)	14.2 ± 5.3	13.1 ± 5.8	14.6 ± 4.9	0.11
Inhospital death (%)	0 (0.0)	0 (0.0)	0 (0.0)	—

Values display total number of patients reported as mean ± SD.

CM: conventional MIMVS; DCT: drawer-case technique

The usage of retrograde cardioplegia was significantly more common in the DCT than the CM group. The aortic cross-clamping time was relatively shorter in the DCT than the CM group without statistical difference. Postoperative junctional rhythm was observed in 6 patients in the CM group and 21 patients in the DCT group. All patients who were in sinus rhythm preoperatively returned to sinus rhythm. One patient in the DCT group who presented with sick sinus syndrome preoperatively required a permanent pacemaker. The mean postoperative stay was 13.1 days in the CM group and 14.6 days in the DCT group, with no significant difference. There were no in-hospital deaths.

## Discussion

The best possible surgical view is clearly a prerequisite for a successful operation, and the optimal method should be selected according to the characteristics of the case. In addition, the standard mitral valve surgery, which consists of a right mini-thoracotomy and a right-sided left atriotomy, can be challenging in some situations. We believe that the DCT can be an optimal method in these difficult cases.

Generally, when the S–V distance is <60 mm, exposure of the mitral valve through a right-sided left atriotomy becomes challenging due to the limited working space for a left atrial roof retractor. In such cases, a median sternotomy is often selected as an alternative approach.^8,9)^ In our study, 12 patients had an S–V distance of <75 mm, and among them, 3 patients had a distance of <60 mm. Surgical intervention through a right mini-thoracotomy is rarely performed in these latter cases. However, using the DCT, the position of the mitral valve is moved into the right thoracic cavity, away from the narrowest portion, thereby overcoming the limitations associated with a narrow S–V distance. In patients with an extremely narrow S–V distance of <60 mm, we create a working space by lifting the sternum using a wire that is usually used for sternum closure and is threaded through the sternum (**Supplementary Video 1**). With these measures, it is possible to increase the S–V distance by 2–3 cm.

Another challenge in mitral valve surgery via a right mini-thoracotomy is obtaining an optimal surgical view. Even when the mitral valve is visible from the front, insufficient exposure of the posteromedial portion often occurs. To improve visualization, many surgeons use retraction sutures on the posteromedial aspect of the left atrial wall, and it is also common to place a retraction suture directly on the diaphragm and pull it downward.^8)^ However, this technique carries a risk of liver injury.^10,11)^ Therefore, we started performing strong caudal retraction of the pericardium adjacent to the diaphragm to safely depress the diaphragm itself. This maneuver elongates the axis formed by the superior vena cava and inferior vena cava, extending not only the right atrium but also the left atrium located behind it. This improves the poor visual field of the posteromedial half side of the mitral valve.

Moreover, we selected the superior septal approach to further enhance the surgical visual field of the mitral valve. This technique provides excellent visualization of the mitral valve, even in cases with a small left atrium or a counterclockwise-rotated heart. By enabling access to the mitral valve through the right atrium, the superior septal approach also facilitates concomitant tricuspid valve surgery, making such procedures highly feasible and readily accessible (**Supplementary Video 2**).

**Fig. 2:**
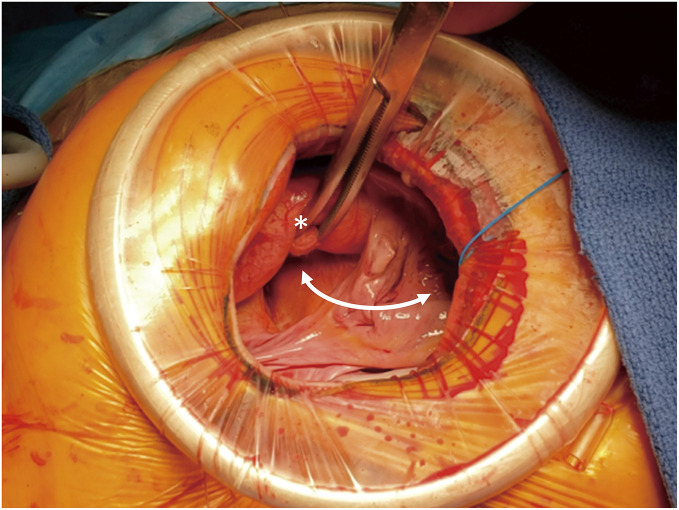
The aortic root and left atrial roof are captured in the center of the surgical field (white arrow: incision line for DCT; white asterisk: pushed up ascending aorta). DCT: drawer-case technique

It is of utmost importance to position the aortic root at the center of the surgical visual field when using the DCT (**Fig. 2** and **Supplementary Video 3**). The aortic root is located immediately below the surgical wound, and the ceiling of the left atrium is located beneath the aortic root; further inside lies the mitral valve. This anatomical relationship not only facilitates access to the mitral valve but also makes concomitant interventions involving the aortic valve—typically challenging to approach simultaneously—more feasible.^12–15)^ In this study, AVR was performed in only one patient in the CM group, whereas 4 patients in the DCT group underwent AVR (**Supplementary Video 4**).

Because of the excellent visualization achieved in the DCT group, we were able to perform not only AVR but also various concomitant procedures such as Morrow myectomy, ventricular septal defect closure, and left atrial plication. As the number of concomitant procedures increases, the aortic cross-clamping time is extended. However, given that access to the heart’s chambers is achieved via the right atrium when using the DCT, the use of not only anterograde but also retrograde myocardial protection is remarkably straightforward. The use of both anterograde and retrograde myocardial protection was significantly more common in the DCT than the CM group [95 (88.0%) vs. 6 (12.8%) patients, respectively]. The DCT is superior from the perspective of ensuring adequate myocardial protection and preventing air embolism. Notably, however, in 3 patients from the DCT group, coronary sinus injury was observed during insertion of the retrograde myocardial protection cannula. A modification was subsequently made to insert the cannula with the rigid stylet removed and place it shallowly into the coronary sinus ostium.

The disadvantages of this method include the need for extended incisions traversing both atria and the potential risk of sinus node dysfunction. Although no significant difference was observed between the 2 groups in this regard in our study, the impact of transecting the sinoatrial node artery (SANa) warrants thorough discussion. It is generally accepted that the superior septal approach offers better exposure of the mitral valve compared with the right-sided left atriotomy or the transseptal approach; however, the risk of postoperative sinus node dysfunction after the superior septal approach remains controversial.^5–7)^ Given the anatomical proximity, it is challenging to avoid injuring the SANa during the superior septal approach, and there is a consensus that this may temporarily result in sinus node dysfunction. Nevertheless, because most patients recover normal sinus rhythm within approximately one week, careful postoperative monitoring should be sufficient to address this concern. We routinely attempt to avoid complete damage to the SANa by directing the incision toward the left superior pulmonary vein. As a result, no persistent junctional rhythm was observed in this study, and all patients who were in sinus rhythm preoperatively had recovered sinus rhythm by the time of discharge. However, there were 2 exceptions in patients with preoperative bradycardia, in whom recovery to sinus rhythm required 14 and 17 days, respectively. Therefore, this approach may be inappropriate for patients with significant preoperative bradycardia.

Although there was no significant difference in the mean postoperative hospital stay between the 2 groups, our institution generally allows for a longer postoperative rehabilitation period, which may have contributed to this result. Therefore, in institutions that implement early discharge protocols, such as discharge within one week after surgery, a difference between the groups may become apparent. There were no cases of readmission for bradyarrhythmias, including junctional rhythm, within 30 days post-discharge.

### Limitations

Our study had several limitations. It was based on a retrospective analysis of our institutional, observational, prospectively collected database, and we were unable to account for the influence of any residual unmeasured factors that could have contributed to adverse outcomes. However, despite the presence of many potential biases, the patients’ baseline characteristics were similar in both groups. This was probably related to the similar selection of patients undergoing mitral valve surgery through a small right thoracotomy

## Conclusion

No significant differences in perioperative factors were observed between the right-sided left atriotomy approach and the superior septal approach for mitral valve surgery via right mini-thoracotomy. Although previous reports have suggested that the superior septal approach may increase the risk of junctional rhythm and permanent pacemaker implantation, our study did not find any significant differences between the 2 approaches.

Conversely, in cases requiring complex procedures—such as double- or triple-valve surgery or valve repair for active infective endocarditis—the superior septal approach offers clear advantages, including enhanced surgical exposure, a wider working space, and the possibility of utilizing retrograde myocardial protection. Therefore, the superior septal approach should be considered an attractive and viable option in these challenging cases.

## Acknowledgments

We thank Angela Morben, DVM, ELS, from Edanz (https://jp.edanz.com/ac) for editing a draft of this manuscript.

## Declarations

### Ethics approval and consent to participate

This retrospective study protocol was approved by our institutional ethics committee (reference number: 20150123), and informed consent was obtained from each patient.

### Consent for publication

Informed consent was obtained from each patient.

### Funding

The authors have no funding sources to disclose.

### Data availability

Underlying data will be made available from the corresponding author upon reasonable request.

### Authors’ contributions

All authors have read and approved the final version of the manuscript.

MY: Project administration; conceptualization; data curation; formal analysis; investigation; methodology; writing—original draft; writing—review and editing. YM: Conceptualization; project administration; data curation; methodology; validation. TT: Visualization. HH: Visualization. NK: Visualization. KH: Conceptualization; methodology; supervision; writing—review and editing. HS: Conceptualization; methodology; supervision; writing—review and editing.

### Disclosure statement

The authors have no conflicts of interest to disclose.

## Supplementary Materials

Supplementary Video 1 Lifting the sternum using a wire that is usually used for sternum closure.

Supplementary Video 2 Mitral valve repair + tricuspid annuloplasty by DCT approach.

Supplementary Video 3 Aortic root and left atrial roof are captured in the center of the surgical field.

Supplementary Video 4 Mitral valve repair + aortic valve replacement by DCT approach.
